# Leveraging the Food System in the Eastern Mediterranean Region for Better Health and Nutrition: A Case Study from Oman

**DOI:** 10.3390/ijerph17197250

**Published:** 2020-10-04

**Authors:** Ayoub Al-Jawaldeh, Salima Almamary, Lamia Mahmoud, Lara Nasreddine

**Affiliations:** 1 World Health Organization (WHO), Regional Office for the Eastern Mediterranean (EMRO), 7608 Cairo, Egypt; aljawaldeha@who.int; 2 Nutrition Department, Ministry of Health, 393 Muscat, Oman; dr.salima.almamary@gmail.com; 3 Office of the WHO Representative, 393 Muscat, Oman; mahmoudl@who.int; 4 Nutrition and Food Sciences Department, Faculty of Agriculture and Food Sciences, American University of Beirut, 11-0236 Beirut, Lebanon

**Keywords:** food system, diet, nutrition, Eastern Mediterranean Region, Oman

## Abstract

The adoption of a food system approach is vital for the Eastern Mediterranean Region (EMR) in achieving the 2030 Agenda. The objective of this paper is to present a case-study from Oman, where a roadmap of context-specific entry points within the food system was proposed, with the overarching aim of fostering healthier diets in the population. A four-staged process was adopted: (1) selection of potential target food groups; (2) assessment of self-sufficiency and sustainability considerations related to the target foods; (3) characterization of challenges, opportunities and potential interventions related to the target food groups and (4) identification of specific entry points within the three elements of the food system (food supply chain; food environment; and consumer behavior). Data collection was based on a review of pertinent literature as well as a participatory approach involving policy makers and stakeholders. Findings showed that fruit, vegetables, fish and foods that are high in fat, sugar and salt are priority targets for intervention. Specific entry points within the food system were identified and a realistic roadmap of activities was outlined. Findings and recommendations presented in this paper may facilitate policy convergence efforts in Oman and serve as a case-study for other EMR countries.

## 1. Introduction

The need for better nutrition was recognized in Goal 2 of the United Nations’ Sustainable Development Goals (SDGs), which aims to “end hunger, achieve food security and improved nutrition, and promote sustainable agriculture” [1]. Adequate nutrition is also recognized as an essential component for achieving many of the other SDGs [2]. Poor nutrition may result from several socio-economic, personal or environmental factors, including food insecurity related to poverty and war, high intakes of processed foods that are low in nutrients and unbalanced dietary patterns [2].

The Eastern Mediterranean region (EMR) harbors countries of different income and development levels, with some Member States witnessing wars and refugees’ crises while others experience economic growth and prosperity. It has been described as a region that hosts extremes in populations’ nutritional status, whereby obesity and diet-related diseases plague some of its countries while others are afflicted with a high burden of undernutrition and food insecurity [3]. Of more concern is the fact that, in many of the EMR countries, different forms of malnutrition tend to co-exist within the same country or community, and often within the same household or individual, highlighting the paradoxical interlinkages that may exist between these forms of malnutrition [4]. In fact, the nutrition situation in many of the region’s Member States can be characterized by a double burden of malnutrition referring to the co-presence of obesity and undernutrition [5]. 

The High Level Panel of Experts (HLPE) on Food Security and Nutrition has highlighted that malnutrition in all of its forms will not be “self-corrected” by economic growth alone, as previously thought, nor will it be addressed by sporadic and punctual nutrition interventions [6]. On the contrary, nutrition ought to be acknowledged and integrated as an explicit objective within national policies, and cross-sectoral nutrition strategies ought to be developed and implemented at different levels in order to optimize impact and effectiveness. The HLPE emphasized that malnutrition cannot be efficiently addressed without “sustainable food systems that facilitate healthy and sustainable food choices” and ensures nutrition security and adequacy for all, including vulnerable population groups [6]. The UN Decade of Action on Nutrition, initiated in April 2016, builds heavily on food systems, calling for food systems that are more nutrition-focused and environmentally friendly.

As defined by the HLPE on Food Security and Nutrition, a food system gathers all the elements (environment, people, inputs, processes, infrastructures, institutions, etc.) and activities with respect to the production, processing, distribution, preparation and consumption of food, and the potential outputs of these activities, including socio-economic and environmental outcomes, as well as nutrition and health outcomes [6]. The conceptual framework presented by the HLPE identifies three constituent elements of food systems that are considered as entry and exit points for nutrition: (1) food supply chains; (2) food environments; and (3) consumer behavior. The food supply chain comprises all activities that move food from production to consumption [6]. It includes food production, storage, distribution, processing, packaging and retailing as well as marketing [6]. It is acknowledged that decisions and choices made at any stage of the food supply chain may have important implications for other stages within the food system [6]. They in fact determine the types of food that are available and accessible, as well as the way these foods are produced and consumed [6]. The food environment refers to the physical, economic, political and socio-cultural context in which the consumer engages with the food system to obtain, prepare and consume food [6]. It is comprised of “food entry points”, such as the physical spaces where food is acquired, the built environment that permits the consumer to access these spaces, the personal determinants of food choices (which include income, education, skills), as well as the political, social and cultural norms that lie beneath these interactions [6]. Within the food environment, the main elements that affect food choices and diets comprise the physical and economic access to food (proximity and affordability), food promotion, advertising and information and food quality and safety. The third constituent element of the food system is consumer behavior, which reflects the choices made by the consumer on what food to obtain, store, prepare and consume, and on the allocation of food within the household [6]. Consumer behavior is undoubtedly determined by personal preferences such as taste, convenience and culture but is also influenced by the prevailing food environment [6]. Food systems have the potential to improve human health and promote environmental sustainability; however, they are currently posing a global risk to both people and the planet [7]. Unhealthy diets are associated with an increase in the burden of non-communicable diseases worldwide and the effects of food production on greenhouse-gas emissions, nitrogen and phosphorus pollution, biodiversity loss and water and land use are compromising the stability of the Earth’s system. Transformation to healthy diets from sustainable food systems is, therefore, crucial to achieve the UN Sustainable Development Goals, and this will require commitment and concerted action from individuals and organizations working in all sectors and at all scales [7]. 

Awareness of the need to transform food systems for better nutrition has increased in the EMR during the past decade. The WHO Regional Strategy (2020–2030) for the EMR focuses on strengthening food systems to tackle the multiple burdens of malnutrition and achieving the 2030 Agenda [8]. Within such a perspective, there is a need for effective policies and strategies that have the potential to shape food systems and contribute to enhanced food security and nutrition. The planning for a food system intervention in any country should be guided by a thorough mapping of the local context to allow for evidence-based prioritization of potential interventions within the food system. In this context, the objective of this paper is to present a case-study from Oman, a country from the EMR [9], where a food system intervention was proposed, with the overarching aim of fostering healthier diets and reducing the double burden of malnutrition. The paper presents a systematic approach for the identification of priority interventions within the food system in Oman and provides a roadmap of context-specific entry points within the three elements of the food system.

## 2. Materials and Methods

**Figure 1:**
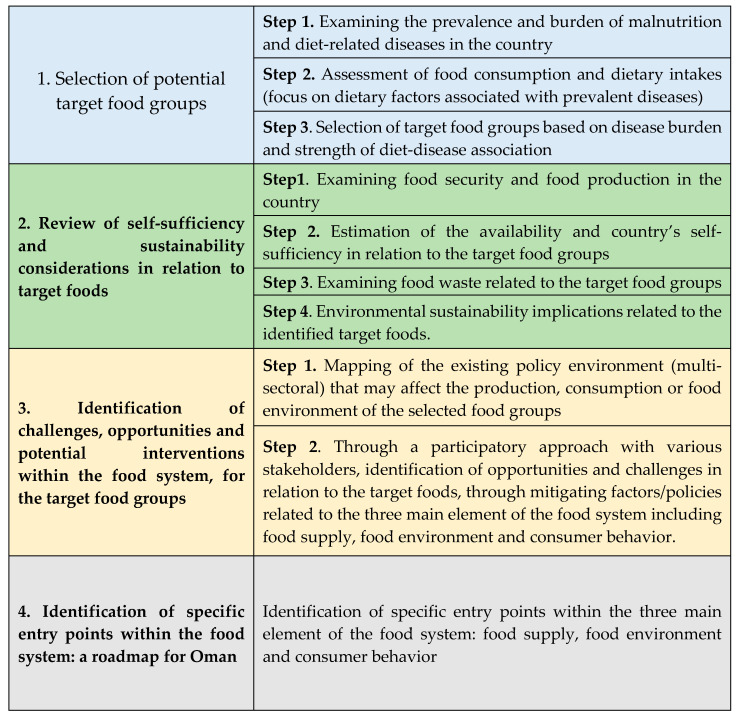
A four staged process to identify priority foods and interventions within the food system.

In this paper, a systematic four-staged approach was adopted for the development of the roadmap and the identification of specific entry points within the food system in the Sultanate of Oman, a country with a population of 4,617,927, a median age of 29.3 years [10] and a Gross Domestic Product (GDP) of USD 76.983 [11]. The adopted approach is presented in Figure 1.

An important pre-requisite for the development and implementation of strategies aimed at improving the nutritional status of the population is a thorough and evidence-based understanding of the current nutrition situation in the country. Thus, the first step in stage 1 was to examine the prevalence and burden of malnutrition and diet-related diseases in the country. This was followed by an evaluation of dietary practices that can be linked to the current burden of malnutrition and chronic diseases. The synthesis of information stemming from these two steps allowed for the identification and selection of potential foods to be targeted by the food system intervention in Oman. Once identified, stage 2 of the process was implemented. Acknowledging the overlap between food systems and agriculture in the area of food production, and acknowledging the role of sustainable food systems in supporting food security [12], step 1 of stage 2 consisted of reviewing pertinent evidence on food security and local food production in the Sultanate. Steps 2–3 assessed the country’s self-sufficiency, food waste and availability with regards to the target food products and examined whether their food supply chain would incur additional environmental constraints. Stage 3 involved the identification of local opportunities and challenges related to the identified target food groups and characterized the existing policy environment that may affect the food supply chain, food environment or consumer behavior in relation of the target food groups. The information stemming from the first 3 stages of the process allowed us to identify realistic, feasible and context-specific points of entry to leverage the food system in Oman (Stage 4). The collection of information within these four stages was based on two complementary methods: (1) a review of pertinent literature to gain a better understanding of the local context and on (2) a participatory approach based on meetings and interviews with concerned policy makers and stakeholders.

A review of pertinent literature was conducted, including individual studies and review articles published between 1990 and 2016, which reported on the nutrition situation of children, adolescents, and adults in Oman, including under and overnutrition indicators, prevalence of diet-related diseases, food consumption patterns, and dietary intakes. Food security challenges, agricultural self-sufficiency, nutrition policy environment, food availability, and environmental sustainability considerations were also reviewed. The following electronic databases were searched: MedLine, PubMed, Scopus and Google Scholar, using a predetermined list of key terms for the search strategy. Other databases were also searched, including the WHO Global School-based Student Health Survey and the Food and Agriculture Organization of the United Nations-Food and Agriculture Organization Corporate Statistical Database (FAO-UN FAOSTAT) Food Balance Sheets In addition, websites and reports prepared by relevant ministries were reviewed to identify additional data sources. The search was restricted to the English and Arabic languages. 

The participatory approach was based on meetings and interviews with policy makers and stakeholders, which were facilitated by the WHO regional EMR office, the WHO country office in Oman, and the Ministry of Health. During these meetings and interviews, input form the various stakeholders was obtained regarding local challenges, opportunities and feasible potential interventions or activities that can leverage the food system. This exercise led to the identification of specific entry points within the food system in Oman in relation to the identified target foods. The findings were later shared, in a multi-stakeholder meeting, with concerned stakeholders for feedback and additional input. This led to the final set of recommendations with regards to the food system entry points and the roadmap for the country.

## 3. Results

### 3.1. Selection of Potential Target Food Groups

1. Step 1. Examining the prevalence and burden of malnutrition and diet-related diseases in the country.

Like other countries of the EMR, the nutritional profile of Oman is characterized by the double burden of malnutrition, with persistent undernutrition and micronutrient deficiencies, coupled with an increasing burden of overnutrition and associated morbidities.

Current prevalence rates of child stunting, underweight and stunting are still a concern. Compared to the preceding national survey conducted in 2009 which showed that 9.8% of under-five children were stunted, the Omani National Nutrition survey (ONNS) conducted in 2017 showed that the prevalence of stunting increased to 11.4% [13,14,15,16]. This increase is in stark contrast with the Global World Health Assembly Nutrition target for 2025, which stipulates an annual rate of change of −3.9% in the prevalence of stunting [17]. Similarly, the prevalence of underweight increased from 8.6% to 11.2% and that of wasting from 7.1% in 2009 to 9.3% in 2017 [15]. The prevalence of wasting thus continues to exceed the Global Health Assembly Target for wasting (<5%) [17]. Although Oman made significant progress in combatting iodine deficiency and vitamin A deficiency [16,18,19,20,21,22,23,24], the prevalence of anemia remains high, as shown by the recent ONNS. Approximately 24% of under-five children [15] and 28% of women of reproductive age were found to be anemic in 2017 [15]. At the same time, the ONNS showed that 53.4% of under-five children and 41.5% of women of reproductive age had vitamin D insufficiency [15]. 

Concomitantly, the prevalence of overnutrition and associated morbidities is increasing in the Sultanate. In school age children, the prevalence of overweight has almost doubled between 1990 and 2016, while the prevalence of obesity has almost quadrupled, reaching 16.1% in boys and 13.3% in girls [25]. The recent stepwise survey conducted in 2017 documented sharp increases in adult obesity, with prevalence estimates rising from 8.4% to 23.2% in men and from 18.9% to 39.3% in women between 1990 and 2017 [25,26]. The findings of the ONNS showed that 59.2% of women of reproductive age were overweight or obese [15]. The increase in obesity may, at least partially, explain the high burden of non-communicable diseases (NCDs) and metabolic abnormalities in the Omani population. A recent systematic review on NCDs and health equity in the WHO Eastern Mediterranean Region showed that 83% of deaths in Oman are caused by NCDs [27]. A high prevalence of NCD risk factors was also observed, with 40% of the adult population being hypertensive, and approximately one third having high cholesterol, low HDL or high LDL-C [28]. The metabolic syndrome was identified amongst 23.6% of adults in Oman, ranging between 13.5% in the 20–29 years age group, and reaching as high as 39.5% in those aged above 50 years [28].

2. Step 2. Food consumption and dietary intakes in Oman.

The double burden of malnutrition and the NCD epidemic that Oman is witnessing may be linked to the nutrition transition that occurred in the country during the past few decades, with its characteristic shifts in diet and lifestyle. Data on food consumption and dietary intakes in Oman are rather scarce, but available evidence highlights faulty dietary practices, characterized by high intakes of energy-dense, nutrient depleted foods, at the expense of cardio-protective nutrient-rich foods such as fruit and vegetables. A study conducted amongst preschoolers showed that only 48.7% and 59.4% of the children met the minimum recommended intake of fruit and vegetables, respectively [29], while the majority met the minimum recommended intake for meat (72.7%) and grains (86%) [29]. Based on the Global School-based Student Health Survey (GSHS), the percentage of adolescents consuming carbonated soft drinks one or more times/day was estimated at 44% amongst 13–15 year old subjects and at 41% in those aged 16–17 years [30]. In contrast, only 24.7% of 13–15-year-old adolescents reported the consumption of fruit and vegetables at least five times/day [30]. Other studies conducted amongst adolescents showed that only 29% consumed an adequate intake of fruit and vegetables [31], while more than half reported high consumption of sweets, potato fries/chips, and fast food [32]. Although the intake of grains was found to be adequate in the majority of adolescents (88%) [32], more than half (52%–57%) consumed less than three servings of vegetables per day [33], and more than a third consumed less than two servings of fruit per day [33]. In addition, the vast majority of adolescents (76.1% of males and 82.9% of females) had a low intake of milk and dairy products (<2 servings per day), whereas a high proportion (67%–78%) reported the consumption of at least three servings of meat on a daily basis [33]. In adults, the Oman Steps Survey 2017 showed that 63.9% of men and 57.3% of women consumed less than five servings of fruit and/or vegetables on average per day [26], while a high consumption of sugar-sweetened beverages (SSBs) was noted, estimated at approximately 120 g/day [34]. The average consumption levels of fruit and vegetables were almost half of the optimal intakes defined by Afshin et al. [34], estimated at approximately 140 g/day and 220 g/day, respectively [34]. Seafood Omega 3 consumption was reported at around 50 mg/day, being almost 1/4th of the optimal intake level [34].

The intakes of atherogenic nutrients were also found to be high in Oman. A very high fat intake was observed amongst adolescents in Oman (40%–42%) [33]. In adults aged 20 years and above, the average intake of saturated fat was estimated at 10.3% of energy intake (EI), which exceeds the global mean consumption level of 9.4% EI [35]. Similarly, the average intake of trans fatty acids (TFA) was estimated at 1.8% EI, which is higher than the WHO upper limit for TFA (1%) [36] and the global average value of 1.4% EI [35]. Based on the recent 2017 stepwise survey, mean salt intake level was estimated at 8.5 g/day amongst adults aged 18 years and above [26]. Based on a systematic analysis of 24 h urinary sodium excretion and dietary surveys worldwide, sodium intake level was estimated at 3.78 g/day amongst adults aged 20 years and above [37], thus exceeding, by almost 100% the upper level set by the WHO (2 g/day).

3. Step 3. Selection of target food groups based on disease burden and strength of diet–disease association.

The selection of target food groups was guided by findings generated by steps 1 and 2, which identified priority nutrition-related public health issues in Oman and documented faulty dietary practices in the population. The selection of target food groups was also guided by the scientific evidence linking dietary practices to the current burden of malnutrition and chronic diseases, particularly the availability of sufficient data in the literature linking exposure to risk, the strength of the epidemiological evidence supporting the association between risk factor exposure and disease endpoints, and evidence supporting the generalizability of the effects to various populations [38].

**Figure 2:**
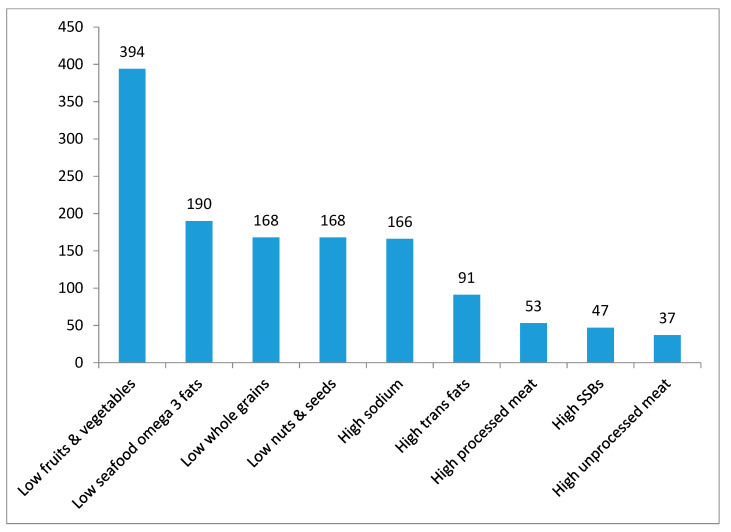
Cardiometabolic deaths (per million adults) attributable to dietary risk factors in Oman (2010).

Available evidence shows that the risk of obesity and NCDs may be modulated by dietary modifications and interventions [39,40]. The WHO recommends people limit energy intake from total fats and sugars, and increase the consumption of low energy, nutrient-dense foods such as fruit and vegetables, as well as legumes, whole grains, and nuts, to decrease the risk of obesity and chronic diseases [39]. The WHO also promotes regular consumption of fish (1–2 servings per week) that are rich in omega-3 fatty acids for the prevention of several chronic diseases [40]. A recent study quantified the impact of suboptimal dietary intakes on NCD mortality in 195 countries. It showed that the leading dietary risk factors for NCD-related mortality are diets high in sodium, and low in whole grains, fruit, nuts and seeds, vegetables, fish and omega-3 fatty acids; each of these dietary factors accounting for more than 2% of global deaths [38]. Another review examining the impact of dietary habits on NCD-related mortality in countries of the Middle East and North Africa showed that the dietary factors that were the highest contributors to cardiometabolic deaths in Oman were low intakes of fruit, vegetables and sea food omega 3 fatty acids and high intakes of sodium, trans fats and sugar-sweetened beverages (SSBs) [34]. As shown in Figure 2, low intakes of fruit (<300 g/day) and low intakes of vegetables (<400 g/day) were found to contribute to 394 deaths per million adults, while 190 cardiometabolic deaths per million adults were attributed to low seafood omega-3 intake (<250 mg/day). In addition, high intakes of sodium (>2000 mg/day), trans fat (>0.5% EI) and SSBs (>0 g/day) contributed together to 304 cardiometabolic deaths per million adults [34]. Mortality related to high intakes of SSBs ranked first in Oman compared to other Gulf Cooperation Council (GCC) countries, and mortality related to high sodium intakes ranked second. It is also important to note that low intakes of fruit, vegetables and fish coupled to high intakes of foods high in fat, sugar and salt (HFSS) may be also contributing to undernutrition, low dietary diversity and micronutrient deficiencies in Oman. The WHO indicates that improving the quality of diets and enhancing dietary diversity are amongst the most effective interventions for the prevention of stunting and anemia in children [41,42,43,44,45]. Increasing the diversity of foods provided to children, particularly animal products such as meat, poultry and fish, in addition to fruit and vegetables, is needed to improve micronutrient intakes and linear growth [46,47,48,49].

Based on data from Afshin et al., 2015 [34]. In this study, vegetables included beans.

Taken together, the review of the burden of malnutrition and diet-related diseases in the Sultanate, the prevalent food consumption patterns and evidence on diet-disease associations led to the selection of the food groups to be targeted by the intervention. Accordingly, fruit and vegetables, seafood, and HFSS are identified as primary targets for intervention in the Omani context. Based on the review by Afshin et al. [34], the total amount of fruit and vegetables available for consumption needs to approximately double in Oman, while the intake of omega-3 fatty acids ought to approximately quadruple [34], in order to meet the recommended amounts for optimal cardio-protective protection. At the same time, the intakes of sodium, trans fatty acids and SSBs should be considerably decreased to offset the harmful health effects of these foods.

### 3.2. Review of Self-Sufficiency and Sustainability Considerations Related to the Target Foods

1. Step 1. Examining food security and food production in the country.

Oman is one of the 72 countries that achieved the Millennium Development Goal (MDG) target of halving the proportion of hungry people by 2015 [50]. This success was the result of commitment and determined action on several fronts: Oman included food security as one of its priorities in the national agenda; it adopted effective measures to boost agricultural productivity and, thus, was able to increase availability and access to food. Extreme poverty was, therefore, reduced in both the urban and rural settings [50]. 

Despite the above, it is important to acknowledge that, similarly to its GCC neighbors, Oman faces constraints to domestic food security, given the challenges imposed by water scarcity, and the limited availability of arable land [51]. Although a comprehensive water management system is in place in Oman, the country faces a constant threat of water shortages. Of Oman’s land (309,500 km^2^), approximately 430,952 hectares is cultivated (≈0.12% of available land), the principal agricultural region being the Batinah Plain in the North. Cultivation depends on irrigation, thus increasing pressure on scarce water resources [51].

The country relies on imports to bridge its food consumption gap [52]; currently, approximately 60% of Oman’s food needs to originate from international markets. In fact, since 2000, the contribution of local agricultural products to total food consumption in Oman has been relatively constant, at around 36% [51]. The sustained capacity of Oman to finance food imports allows it to maintain the population’s food security. However, it renders it vulnerable to potential disruptions of food supply and to price fluctuations on global markets [51]. Needless to say, the demand for food is likely to increase in the coming years as a result of population growth (estimated at 5.3%) [10] and increased wealth [53]. A national committee for food security was established in Oman, responsible for drafting a comprehensive strategy for future food security. A key role in this process is played by the Public Authority for Stores and Food Reserves (PASFR). 

The local agricultural produce of Oman includes mainly dates, limes, bananas, alfalfa, vegetables, camels, cattle and fish. Oman is one of the most important fish producers in the region and has a net export of fish and fish products [54]; around 47% of the fish capture is exported, of which 61% is sold to neighboring GCC states [54]. Total fish capture production was relatively stable between 2005 and 2011 at approximately 155,000 tons per year but has increased since 2012, reaching 206,200 tons in 2013 [54] and 280,000 tons in 2017. There has been an estimated 7% increase in annual production during the past decade. As for agricultural produce, date plantations occupy almost half of the country’s agricultural land; dates’ domestic production meets local demand and generates significant surplus for export [51,52]. The country may benefit from increases in agricultural output by instilling intensive farming practices that are more water efficient and adopting modern techniques and technologies [51]. “Moving towards intensive, productivity-driven improvements” will necessitate a strategy outlining priority crops, based on water and land efficiency, sustainability, profit rates and adaptability to the local context. A potential candidate could be fruit and vegetable cultivation, given that Oman already has relatively high self-sufficiency ratios for these crops and given that fruit and vegetables can be sustainably cultivated, without jeopardizing scarce land and water resources [51].

Although technological advancement may ensure increased production of foods, other options should also be considered, such as modifying food preparation practices and decreasing food waste in Oman [55]. Based on a study conducted at Sultan Qaboos University (SQU), the average Omani family wastes about a third of all food prepared within the household. The highest waste was observed amongst children of ages 1–5 years (57%), and children and adolescents aged 6–18 years (56%), while the lowest was observed amongst adults above the age of 40 years (7%). It was estimated that the average family food waste was 35% by weight [55].

2. Step 2. Estimation of the availability and country’s self-sufficiency in relation to the target food groups.

Available data indicate that fish, fruit, and vegetables have the highest self-sufficiency ratio (100% for fish, 74% for fruit, and 72% for vegetables) in Oman [51], for the current population consumption rates. Needless to say that efforts should be made to sustainably increase national production and self-sufficiency for these products to meet the desired increases in population’s consumption levels, which ought to double for fruit and vegetables, and quadruple for omega-3 fatty acids from fish.

**Figure 3:**
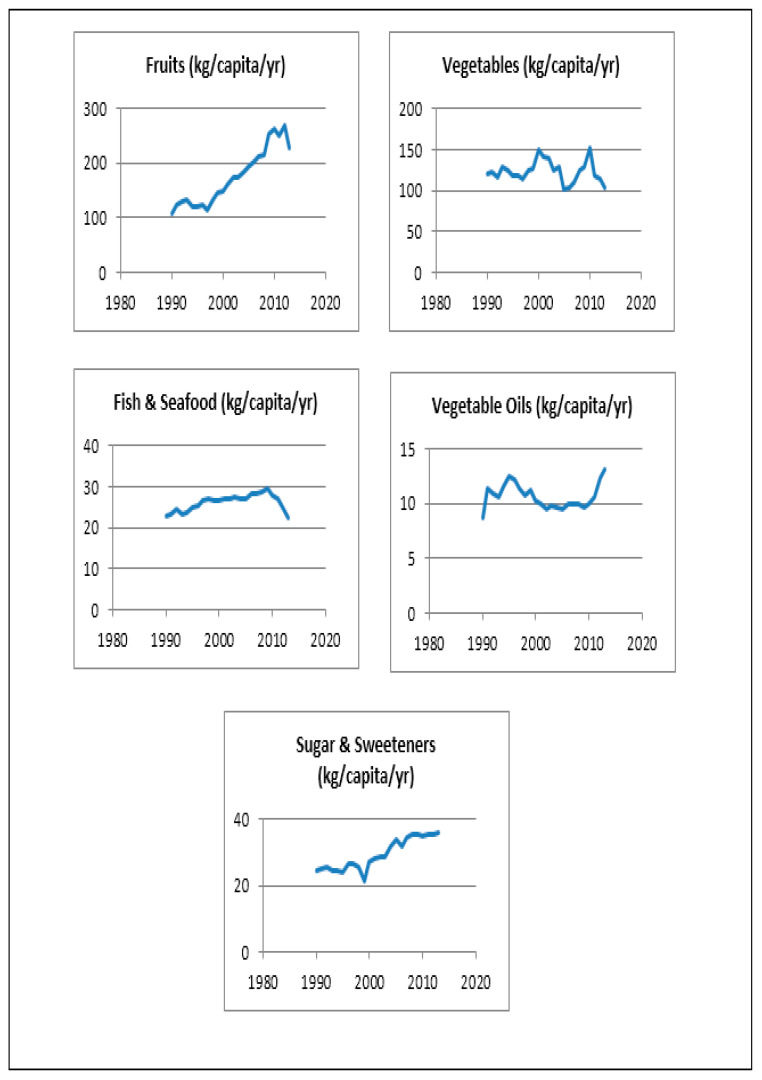
Trend in daily per capita supply (kg) of food groups in Oman. Data from the Food and Agriculture Organization (FAO) food balance sheets, Food and Agriculture Organization Corporate Statistical Database (FAOSTAT) [56].

Figure 3 displays the trend in per capita food supply for the target food groups, based on FAOSTAT data. It shows that, since the 1990s, the per capita supply of fruit has been steadily increasing, the sharpest increases being noted between 2005 and 2010 (from 192 kg/capita/year to 264 kg/capita/year) (Figure 3) [56]. In contrast, the per capita supply of vegetables has remained relatively stable over time, even though local production has almost doubled between 2011 and 2016 [56]. The average annual per capita consumption of fish in Oman has been in the range of 26.7–27.8 kg between 2000 and 2011, highlighting an increase compared to values reported in the 1990s [54]. However, a decreasing trend in fish availability is noticed after 2011. The government aims to enhance fisheries production to reach approximately 480,000 tons in 2020 [54]. Aquaculture production in 2017 was of only 77 tons [54], but there is a high potential for the development of aquaculture in the Sultanate. The availability of sugar has increased by 1.5-fold between 1990 and 2013 (from 24.59 kg/capita/year to 36.05 kg/capita/year) [56]. The per capita supply of vegetable oils in 2013 was reported at 13.07 kg/capita/year, highlighting an increase in its availability compared to 1990 [56].

3. Step 3. Examining food waste related to the target food groups.

It is important to note that fresh produce in Oman experience significant postharvest losses, that can reach up to 19% of perishable commodities [57]. A study of the possible causes behind these losses showed they are mainly attributed to inappropriate harvesting conditions (such as immature or over mature harvesting, direct exposure of commodity to sunlight, inadequate containers in the field, mechanical damage or delays in transportation), inadequate preparation for the market (such as lack of precooling prior to shipment, inappropriate handling, ventilation and cooling or failure to pre-sort), inadequate transport (such as rough handling and improper management of temperature, humidity and ventilation) and improper handling at destination (such as exposure to undesirable environmental conditions, rough handling and improper storage) as well as handling at home (improper storage and delay before consumption) [58]. Data on the causes of losses of fish and the status of the food supply chain in Oman are lacking. It is estimated that losses are mostly caused by poor status of the cold chain infrastructure and unavailability or scarcity of basic hygiene and home level refrigeration facilities [58]. It was also estimated that processing, packaging and distribution are the primordial causes of loss (69%) of fish along the food supply chain in the Middle-East North Africa region, which includes Oman [59].

4. Step 4. Environmental sustainability implications related to the identified target foods.

The EAT Lancet Commission on healthy diets from sustainable food systems proposed a reference dietary pattern that is congruent with human health, a pattern that was also shown to be within environmental boundaries, when combined with improvements in production efficiency and reductions in food loss and waste [7]. The boundaries that were taken into consideration included cropland use, biodiversity loss, water use, green-house-gas emissions, and nitrogen and phosphorous pollution that can result from food production [7]. According to the commission, the transformation to healthy diets by 2050 necessitates substantial dietary shifts, including a greater than 50% reduction in the consumption of unhealthy foods such as high sugar foods, and a greater than 100% increase in the consumption of healthy foods such as fruit and vegetables [7]. Accordingly, the reference diet consisted of specific intake targets for the various food groups; it recommended the intakes of 300 g/day of vegetables and 200 g/day of fruit, or approximately five servings per day. It also provided a possible range of up to 600 g/day for vegetables and up to 300 g/day for fruit. The reference diet also specified about 28 g of fish per day, while also suggesting that the range of possible intake may reach up to 100 g/day [7]. The reference diet also highlights the importance of decreasing sugar and added fats in the diet. Therefore, the recommended interventions to increase fruit, vegetable and fish intake and decrease HFSS consumption take into consideration environmental sustainability implications.

### 3.3. Identification of the Challenges, Opportunities, and Potential Interventions within the Food System, for the Target Foods

1. Step 1. Map the existing policy environment (multi-sectoral) that affect consumption of the selected food groups.

A very active policy response to the nutritional challenges and increasing burden of diet-related diseases is noted in Oman. A National Nutrition Strategy 2014–2050 [60] was developed by the Department of Nutrition at the Ministry of Health, outlining the priority nutrition problems that ought to be tackled in the country. The goals of the strategy target three domains: (1) Health and nutrition; (2) Food security and quality; and (3) Physical fitness through active living. In addition, Oman is one of the few countries in the region that have developed national food-based dietary guidelines (2009) [61]. The *Omani Guide to Healthy Eating* was released in 2009 by the Department of Nutrition (Ministry of Health Oman) to provide dietary advice, which when followed may contribute to the prevention of obesity and chronic diseases in the population [61]. 

The inclusion of the identified target food groups (fruit, vegetables, fish and HFSS) as priorities for intervention is in line with the priorities identified by the National Nutrition Strategy of Oman 2014–2050 and the *Omani Guide to Healthy Eating.* In fact, considering the high burden of cardiometabolic abnormalities and NCDs in Oman, the National Nutrition Strategy of Oman 2014–2050 has emphasized the importance of adequate intake of fruit and vegetables while decreasing the intake of energy dense-foods, as a main determinant of health as well as food security and quality. The strategy includes specific goals related to the promotion of fruit and vegetables at the expense of energy dense foods, such as (1) to increase intake of fresh fruit and vegetables by reducing importation of cardiotoxic processed, high-density foods to <30% of all intake through cooperative agreements with regional suppliers, and increased regulation and import tariffs on non-healthful foods; and (2) to increase local food self-sufficiency and dietary diversity through organically grown vegetables and fruit by introducing high-tech (i.e., nutrient and water efficient) rural and urban gardens in 80% of all households. In addition, the National Nutrition Strategy of Oman 2014–2050 included fish as one of its strategic measures to increase local food self-sufficiency and dietary diversity [60]. 

Increased consumption of fruit and vegetables has also been highlighted as a priority recommendation of the Omani Guide to Healthy Eating, within Guidelines 3 and 4 (Consume 3–5 servings of vegetables daily; Consume 2–4 servings of fruit daily), while the reduction of fat, saturated fats and TFA intakes was emphasized in guideline 8 (Limit your fat intake and choose your snacks wisely) [61]. Increased fish consumption was also included as a priority recommendation of the *Omani Guide to Healthy Eating*, within Guideline 5 (Consume fish, poultry, eggs or lean meat). This guideline clarifies that in Oman, the consumption of fish is much lower compared to the consumption of meat and chicken. For instance, the consumption of fish is five times less than the consumption of chicken [61]. The guideline emphasizes fish as a major source of protein and iron, hence underlining its importance in the prevention of anemia and undernutrition. It also highlights fish as the major source of ω-3 fatty acids, linking the latter to the prevention of hyperlipidemia and cardiovascular diseases. In addition, the strategic plan of the Ministry of Agriculture and Fisheries 2020–2040 indicates that the overall production in Oman will double (and this will be based on deep sea fishing and not artisanal fishing).

In the Sustainable Fishery Development Strategy towards 2030, the Ministry of Agriculture and Fisheries’ Wealth has developed a road map for the completion of the sustainable fisheries and aquaculture development strategy [54]. The strategy indicates that fisheries and agriculture are amongst the oldest and most important production sectors in Oman, highlighting their pivotal role in feeding the population, providing employment opportunities and helping to boost the Sultanate’s GDP [54]. The strategy highlights a strong governmental commitment to develop the fishery sector in a competitive and sustainable manner that is in congruence with the economic, social, cultural and historic values of the country [54]. The strategy builds on the real opportunity to increase capture fisheries production, develop the aquaculture sector and improve the availability of fresh fish in the domestic market [54], while underlining the need for sustainable utilization of the available fish reserves and resource conservation [54]. The overall plan to develop the fisheries sector includes expansion of existing fleet and harbors, development of fish markets, awareness and support to fishermen, aquaculture development and additional fish processing and logistics services.

The Sultanate has also developed and implemented infant and young child nutrition programs [60,61], school feeding programs [23,62], NCD screening programs [60], food labeling regulations [63,64,65] and several initiatives aimed at reducing the intakes of salt, trans fats and saturated fats [66]. A salt reduction strategy was initiated in 2014 and operationalized through efforts by bakeries to reduce salt by 10% and later 20%. In December 2019, a new standard establishing maximum levels for salt in bread was issued, while taxes were introduced on soft drinks (50%), energy drinks (100%) and fast food (5%) as of June 2019. In terms of governance, a multisectoral Nutrition Committee operates under the overarching National Multisectoral NCD Committee, aiming for more coordinated action across all sectors.

However, despite the many existent nutrition programs/policies in Oman—some of which have been running for years (vitamin and mineral supplementation, nutrition education, etc.)—undernutrition and anemia remain a public health concern in the country and the burden of overnutrition, obesity and associated NCDs continues to escalate. This highlights the need for better coordination and collaboration between stakeholders to enhance the effectiveness and impact of the various strategies and policies.

2. Step 2. Through a participatory approach with various stakeholders, identify opportunities and challenges in relation to the target foods.

The identification of appropriate interventions within the country necessitates input from the various stakeholders, regarding feasibility, challenges, opportunities, interest and level of commitment. In this context, meetings and interviews were held with the different stakeholders in Oman, in December 2017, including the Ministry of Health, Ministry of Agriculture and Fisheries Wealth/Planning and Agriculture Studies, Ministry of Agriculture and Fisheries Wealth/Policy and Investment, Ministry of Agriculture and Fisheries Wealth/Marketing and Investment for Agriculture and Livestock, Ministry of Agriculture and Fisheries Wealth/Fisheries Marketing and Investment, Ministry of Commerce/Department of Standards, Ministry of Municipalities/Centre for Food and Water Laboratories, Public Authority of Stores and Food Reserve/Food Security Department, Public Authority of Consumer Protection, Muscat Municipality, Oman Food Investment Holding Co. (OFIC), WHO, FAO and UNICEF.

The inputs provided by the various stakeholders allowed them to map the challenges, opportunities and potential interventions within the food system, as applicable to fruit, vegetables, fish and HFSS, respectively (data not shown). The mapping exercise also showed that several initiatives and projects were being planned or undertaken in Oman, along various elements of the food system, but with limited coordination between the different stakeholders. This identified, therefore, a valuable opportunity in linking these various projects/initiatives under one unified umbrella project to amplify the generated outcomes, while also fortifying the ongoing initiatives with additional interventions that, together, would align the food system towards the main diet quality goals. It is important to note that, in all the meetings that were held with the various stakeholders in Oman, one key theme that was repetitively highlighted was the political commitment and willingness to create an enabling environment for sustainable food systems in the Sultanate.

### 3.4. Identification of Specific Entry Points within the Food System: A Roadmap for Oman

**Figure 4:**
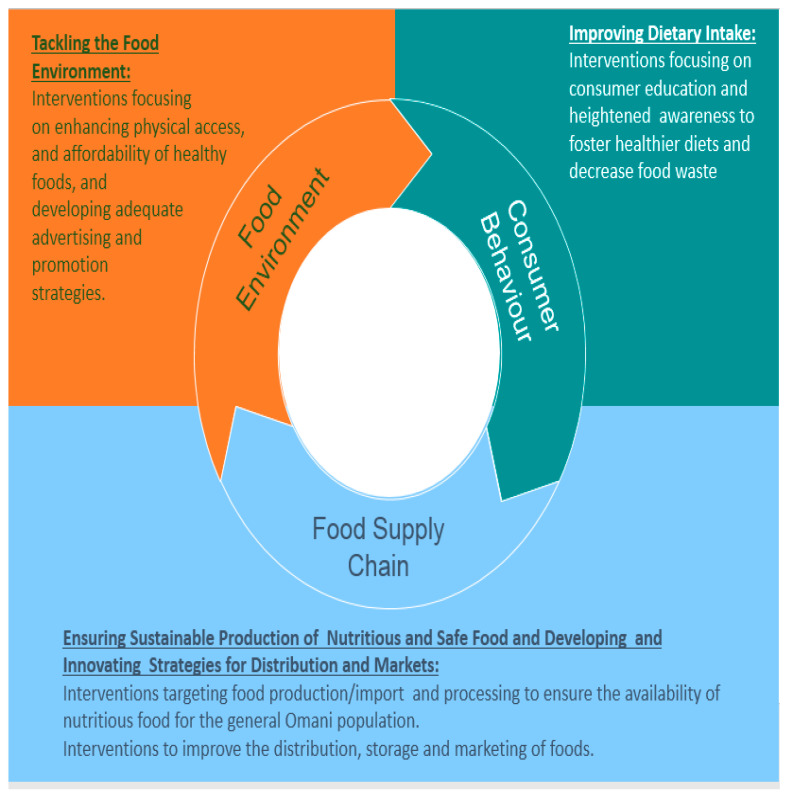
The three constituent elements of food systems and the entry points for intervention [6].

Based on the priorities identified in the three steps summarized above, the following objectives for the intervention were set forth: (1) to increase the population’s consumption level of fruit and vegetables through appropriate interventions in the food system; (2) to increase the population’s consumption level of fish through appropriate interventions in the food system and (3) to decrease the population’s consumption of foods high in sugar, salt and fat (including saturated fat and trans fat) through appropriate interventions along the three elements of the food system (Figure 4).

Specific entry points for intervention within the food system were identified, and a roadmap that responds to the current challenges and opportunities in Oman, and reflects the feedback and input provided by the various stakeholders and policymakers, was developed. The proposed roadmap is shown in Table 1 and includes a range of activities that are mapped along the various components of the food system as illustrated in Figure 4: (i) Food supply chain (food production, storage, processing, and distribution); (ii) food environment (physical access, availability, advertising, affordability) and (iii) consumer behavior (education and awareness). The proposed roadmap is supported by current evidence showing that a package of policies and interventions that target a wide range of interconnected factors, such as food production, processing and environment, in addition to consumer behavior, are more effective than those targeting consumer education alone, in improving the population’s diet [6,38]. The proposed roadmap also builds on existing evidence underlining the effectiveness of population-level dietary interventions such as food pricing strategies (subsidies and taxation), school procurement policies, worksite wellness programs and mass-media campaigns in fostering positive changes in dietary habits [67,68,69].

For fruit, vegetables and fish, and within the food production component of the food supply, activities will focus on diversifying and increasing the production of nutrition-sensitive agricultural crops and further developing the fishery sector (Table 1). These activities will build on existing initiatives that aim at enhancing the production of agricultural crops and fish in the Sultanate, using innovative and sustainable production techniques. Examples of such initiatives include the Integrated Farms Project, the School garden project, the Home Garden program and the development and expansion of the fisheries’ sector. Currently, these initiatives operate independently, which may compromise their nutritional impact. Hence, within the food production component, efforts will be dedicated to optimizing existing initiatives under one common umbrella. In this context, three main initiatives are identified: (1) Integrated farms project that is planned by the Ministry of Agriculture and Fisheries; (2) School garden project that is planned by Ministry of Education and the Ministry of Agriculture and Fisheries in collaboration with Oman Food Investment Holding Co. (OFIC), and (3) Home garden program that is planned by Public Authority for Investment, Promotion and Expert development (*Ithraa*). Activities will also consist of establishing links between production, adequate post-harvest management, and transportation solutions (such as unbroken cold chain). Within the storage, processing and distribution elements of the food system, the planned activities will consist of establishing links between production (vegetables and fish), adequate post-harvest management and transportation solutions (such as unbroken cold chain), to minimize waste, maintain the quality of produce and enhance its distribution. In this respect, efforts will be dedicated to optimizing existing initiatives, such as the Ministry of Agriculture and Fisheries’ (MAF’s) new integrated farm project (which includes the development of postharvest infrastructure) and to provide proper training of farmers. Activities within the food environment will focus on the school feeding program, the school canteen, and public institutions’ procurement, to increase access to fruit, vegetables and fish and decrease access to HFSSs. In addition, activities pertinent to the promotion of fruit, vegetables, and fish as “local healthy” Omani products in supermarkets as well as enhancing their product placement will be conducted. Within the consumer behavior component, activities aiming at raising nutritional awareness and highlighting the importance of fruit, vegetables and fish as part of healthy diets will be conducted at various levels: (1) schools; (2) community and (3) media campaigns. For HFSS, and within the food production element, proposed actions include to legislate and incentivize the food industry to produce healthier food products with less added sugar, salt, and fat. Interventions pertinent to HFSSs will also focus on the development of nutritional standards, the establishment of proper monitoring and the enforcement mechanisms, expanding taxations, removing subsidies, regulating the marketing of HFSSs to children and adopting consumer-friendly labeling systems, all of which are policies that ought to be implemented nationwide. 

Given the plethora of activities/actions that will be conducted to tackle the different entry points in the food system, there is a crucial need for effective coordination between the various stakeholders. This can best be achieved by establishing a national multisectoral committee that represents all key government and private stakeholders, and that aligns the different national policies towards the main diet quality goals. The establishment of the national committee will foster strategic planning, collaboration, coordination and communication between the various amongst stakeholders, across the food system.

## 4. Strengths and Limitations

The strengths of this study include the adoption of a systematic approach for the identification of priority interventions within the food system, and the use of two complementary methods in data collection, which included (1) a thorough review of pertinent literature and (2) a participatory approach involving policy makers and stakeholders. Given that food system interventions ought to be anchored within the local context [70], this study was based on a thorough understanding of the nutrition situation and food consumption patterns in Oman. It is, however, important to mention that the latter may have been limited by the scarcity of national food consumption surveys in the population and the lack of national food composition databases, which limit the accurate estimation of nutrients of potential public health concern, particularly from traditional recipes and mixed dishes [38,71,72]. Other limitations of this study are related to the criteria adopted for the selection of the target foods, and which were based on the availability of sufficient data in the literature linking dietary exposure to risk [38]. This approach may have led to the exclusion of traditional, locally consumed food products that may be also associated with nutritional and health benefits, but for which the epidemiological evidence is still scarce. Finally, one of the pivotal elements in our approach was the stakeholder engagement, whereby representatives from the public sector were consulted for the identification of appropriate and feasible interventions in the country, appraisal of governmental commitment toward such interventions, and evaluation of existent challenges and opportunities. This process was restricted to the public sector and future work should engage other stakeholders, including consumers’ associations, the Omani farmer association, fishermen representatives, women’s associations and youth clubs. 

## 5. Conclusions

This paper is the first from the EMR to plan for a national food system intervention that is guided by a thorough understanding of the local context and an evidence-based prioritization of potential activities and policies within the food system. In order to foster healthier diets in the Omani population, this study adopted a systematic four-staged approach consisting of the (1) selection of target foods based on the local nutrition situation (2) examination of the country’s self-sufficiency in relation to the target foods while considering sustainability implications, (3) identification of challenges, opportunities and initiatives for the target food groups and (4) finally the identification of specific entry points within the food system. This process led to the development of a realistic roadmap of activities that are mapped along the three elements of the food system. The proposed roadmap provides a practical set of specific interventions and activities that can be taken by farmers, food-sector entrepreneurs (suppliers, processors, distributors), consumers, community members, governmental bodies, policy makers and other relevant stakeholders to create a local food system that reflects Oman’s aspirations for better nutrition, increased sustainability and self-reliance. The findings and recommendations presented in this paper will facilitate policy convergence efforts in Oman and guide many stakeholders in contributing to progress towards more sustainable food systems and enhanced food security and nutrition in the country. The approach adopted in this paper may serve as a case-study for other countries in the EMR.

## Figures and Tables

**Table 1:** Identification of specific entry points within the food system: a roadmap for the Sultanate of Oman.

	Food Supply Chain	Food Environment	Consumer Behavior
**Fruit, Vegetables and Fish**	*Food Production*: • Strengthen agricultural production towards the best sustainable ecosystem practices. • Build on the “Integrated Farms Project” that is planned by the Ministry of Agriculture and Fisheries (MAF). The project integrates aquaculture and vegetable production, focuses on small and medium enterprises (SMEs) and involves a public-private partnership. The integrated farm model will entail sustainable production of fish and vegetable crops in the integrated farms. • Implement and expand the “School Garden Project that is developed by Ministry of Education and MAF in collaboration with Oman Food Investment Holding Co. (OFIC). The program will focus on vegetable production within schools. • Implement and expand the “Home Garden Program” that is planned by the Public Authority for Investment, Promotion and Expert development (*Ithraa*). The program focuses on vegetable production within home gardens. It would be recommended to expand it to a homestead production model that also integrates other food production aspects such as aquaculture. • Development of aquaculture and expansion of harbors and fleet. *Storage, processing and distribution:* • Establish links between production (vegetables and fish), adequate post-harvest management and transportation solutions (such as unbroken cold chain), to minimize waste and maintain the quality of produce and enhance its distribution. • Optimize existing initiatives, such as MAF’s new integrated farm project (which includes the development of postharvest infrastructure). • Provide proper training of farmers and fishermen. • Build on existing initiatives such as the integrated fish processing facilities (facilities for seafood processing and packaging, facilities for storing raw materials and deep freezers for exports).	• Revise the 2017 school feeding program to enhance the nutritional quality of foods offered to children in schools. • Enhance access of children and adolescents to fruit, vegetables and fish. • Link school provisions to local farms (for instance, foster linkages with the Integrated Farms’ Project” launched by MAF). • Enhance the palatability and attractive packaging of healthy food options offered in schools. • Implement and enforce the school canteen regulations. • Link the production of vegetables, fish and fruit (including dates) to public institutions’ procurement. • Promote fruit, vegetables and fish as “local healthy” Omani products in supermarkets (local products’ branding). • Enhance product placement of these foods in supermarkets. • Development of fish markets.	• Revision of school curricula to incorporate education on food, diet, and health in schools: integration into and across the curriculum. • Integration, within the curriculum, of hands-on experiential learning. • Education on food waste prevention (experiential learning). • Training of teachers on healthy diets and ways to incorporate fruit, vegetables and fish in the diet. • Dissemination (and revision) of Food-based dietary guidelines. • Development of culture-specific nutrition education material. • Social marketing of key educational messages. • Media campaigns. • Nutrition education and awareness activities targeting women’s associations (as agents of change). • Establishment of community kitchens. • Participation in local festivals to promote the consumption of fruit, vegetables and fish.
**Foods High in Fat, Sugar and Salt (HFSS)**	• Develop standards and regulations for salt, sugar, saturated fat and total fat in food products to serve as a benchmark for product reformulation in the food industry and for food imports. • Legislate and incentivize the food industry to produce healthier food products with less added sugar, salt and fat: Incentivize product reformulation and enforcement of standards. • Establish proper monitoring and enforcement mechanisms to foster adherence to TFA standards in processed foods (in both imported and locally produced foods). • Replace palm oil by healthier types of oil in the food industry.	• Revise the 2017 school feeding program to enhance the nutritional quality of foods offered to children in schools. • Decrease the access to and availability of energy-dense nutrient-depleted foods and beverages in schools. • Expand the taxations that were implemented on soft drinks to all other SSBs while further increasing the taxation magnitude to 100%. • Increase the taxation that was implemented on fast food (5%) to at least 50%. • Implement the traffic light labeling system, for prepacked foods. • Assess the consumer’s subjective and objective understanding of the traffic light system. • Revise the food subsidy program (currently palm oil and sugar are subsidized). • Regulate the marketing of foods and beverages high in sugar, fat, saturated fat, trans fat and salt to children in local media, schools, billboards, events etc. • Adopt the EMR nutrient profile to restrict the marketing of HFSS. • Regulate restaurants and convenience stores around schools.	• Revision of school curricula to incorporate education on food, diet and health in schools: integration into and across the curriculum. • Training of teachers on healthy diets and ways to identify and limit the consumption of HFSS. • Dissemination (and revision) of Food-based dietary guidelines. • Assessment of the determinants of high fat, high sugar, and high salt intake in the Omani population to identify gaps in knowledge and unfavorable attitudes in different age groups. • Use of the data stemming from the knowledge and attitude assessment to develop culture-specific media campaigns.
